# The effect of intermittent fasting on preventing obesity-related early aging from a molecular and cellular perspective

**DOI:** 10.25122/jml-2023-0370

**Published:** 2024-03

**Authors:** Nurma Yuliyanasari, Purwo Sri Rejeki, Hanik Badriyah Hidayati, Phawinee Subsomwong, Muhammad Miftahussurur

**Affiliations:** 1 Doctoral Program of Medical Science, Faculty Of Medicine, Universitas Airlangga, Surabaya, Indonesia; 2 Department of Physiology, Faculty of Medicine, Universitas Muhammadiyah Surabaya, Surabaya, Indonesia; 3 Physiology Division, Department of Medical Physiology and Biochemistry, Faculty of Medicine, Universitas Airlangga, Surabaya, Indonesia; 4 Department of Neurology, Faculty of Medicine, Dr. Soetomo Teaching Hospital, Universitas Airlangga, Surabaya, Indonesia; 5 Department of Environmental and Preventive Medicine, Faculty of Medicine, Oita University, Yufu, Japan; 6 Division of Gastroenterology-Hepatology, Department of Internal Medicine, Faculty of Medicine, Dr. Soetomo Teaching Hospital, Universitas Airlangga, Surabaya, Indonesia; 7 *Helicobacter pylori* and Microbiota Study Group, Institute of Tropical Diseases, Universitas Airlangga, Surabaya, Indonesia

**Keywords:** intermittent fasting, aging, obesity, human health, ADF, alternate-day fasting, ADMF, alternate-day modified fasting, AMPK, AMP-activated protein kinase, β-HB, β-hydroxy butyric acid, BMI, body mass index, IF, intermittent fasting, FOXO, fork head box O, IIS, insulin/insulin-like growth factor signaling, mTOR, mammalian target of rapamycin, PF, periodic fasting, PGC-1α, peroxisome proliferator-activated receptor gamma coactivator 1-alpha, PI3K, phosphatidylinositol 3-kinase, TRE, time-restricted eating

## Abstract

Obesity is a global health concern owing to its association with numerous degenerative diseases and the fact that it may lead to early aging. Various markers of aging, including telomere attrition, epigenetic alterations, altered protein homeostasis, mitochondrial dysfunction, cellular senescence, stem cell disorders, and intercellular communication, are influenced by obesity. Consequently, there is a critical need for safe and effective approaches to prevent obesity and mitigate the onset of premature aging. In recent years, intermittent fasting (IF), a dietary strategy that alternates between periods of fasting and feeding, has emerged as a promising dietary strategy that holds potential in counteracting the aging process associated with obesity. This article explores the molecular and cellular mechanisms through which IF affects obesity-related early aging. IF regulates various physiological processes and organ systems, including the liver, brain, muscles, intestines, blood, adipose tissues, endocrine system, and cardiovascular system. Moreover, IF modulates key signaling pathways such as AMP-activated protein kinase (AMPK), sirtuins, phosphatidylinositol 3-kinase (PI3K)/Akt, mammalian target of rapamycin (mTOR), and fork head box O (FOXO). By targeting these pathways, IF has the potential to attenuate aging phenotypes associated with obesity-related early aging. Overall, IF offers promising avenues for promoting healthier lifestyles and mitigating the premature aging process in individuals affected by obesity.

## INTRODUCTION

Aging is a biological process marked by worsening health, changed metabolism, a functional decline of tissues and organs, structural deterioration, and diminished adaptability [1,2]. One of the many factors that can cause aging to manifest more quickly is obesity [3]. Obesity can cause early aging due to metabolic imbalances, as well as cellular and biomolecular changes [3]. Potentially, obesity can create an environment that increases cell senescence [4].

Over 800 million individuals worldwide suffer from obesity, which is expected to reach epidemic proportions by 2030, affecting over 1 billion persons [5]. It is believed that this will also increase the prevalence of non-communicable diseases, which are currently responsible for 71% of all deaths worldwide [6].

Numerous therapeutic strategies aim to address both obesity and promote healthy aging, with dietary modifications such as intermittent fasting (IF) emerging as a simple and safe approach [7–10]. IF encompasses various eating patterns known to facilitate healthy aging, promote longevity, and sustain overall health [9,11], and the ketone bodies produced during IF improve the body’s bioenergetics and metabolic efficiency [9]. Although previous research has explored the effects of IF on aging, its impact on obesity-induced early aging, particularly in humans, remains to be fully elucidated. The aim of this study was to review the potential of IF in preventing obesity-related early aging from a molecular and cellular perspective.

### The hallmarks of aging

Aging is characterized by the progressive decline of physiological integrity, resulting in diminished function and an increased vulnerability to mortality. Its defining characteristics include genomic instability, telomere attrition, epigenetic modifications, altered protein homeostasis, dysregulation of nutritional signaling, mitochondrial failure, cellular senescence, stem cell dysfunction, and impaired intercellular communication [12]. These hallmarks represent the biological mechanisms dictating the pace of aging and can be categorized into three classes: primary, antagonistic, and integrative hallmarks [1,12].

Genomic instability, telomere attrition, epigenetic changes, and loss of proteostasis are the defining characteristics of the most prevalent sources of damage [12–14]. In vitro and in vivo, DNA damage and mutations that cause genomic instability are crucial indicators of aging cells [14]. Somatic mutations, improper clonal proliferation, DNA modification and repair mechanisms, cellular checkpoint responses, replication fidelity integrity, and antioxidant systems that can repair DNA are all recognized effects of genome instability [1,12]. In the germline, stem cells, and mitotic cells, telomere attrition results in dysfunction. Maladaptive epigenetic changes involve inappropriate DNA methylation at specific sites and specific histone modifications. Reduced protein function, increased levels of damaged or misfolded proteins, and the persistence of non-recycled proteins and organelles are all consequences of proteostasis dysfunction during aging [1,12–14].

The dysregulation of nutritional signaling is an essential hallmark of aging. It is initially a compensatory response that can reduce damage, but it eventually causes further damage in chronic or severe conditions [1,12]. The insulin/insulin-like growth factor-1 (IGF-1) signaling (IIS) pathway becomes more active when nutritional signaling is dysregulated with aging. The role of anabolic signaling in accelerating aging is strongly supported by the increased activity of the mammalian target of rapamycin (mTOR) pathway [1,12].

The mitochondrium is the primary energy source of cells and is involved in many cellular processes, including cell metabolism, inflammation, and cell cycles. However, mitochondrial dysfunction is also one of the critical hallmarks of aging [15]. This condition can disrupt mitochondrial biogenesis, recycling, and disorganization, leading to increased levels of reactive oxygen species (ROS) and respiratory system imbalance [12,16,17].

Cellular senescence is intricately linked with aging and includes the inability to proliferate, loss of normal cell function, secretion of proinflammatory factors, alteration of neighboring cell behavior, and protease-mediated degradation of extracellular components, all of which will cause biological dysfunction [4,12,13]. Senescent cells exhibit a distinctive pathogenic senescence-associated secretory phenotype, which induces secondary senescence, disrupts tissue homeostasis, and impairs tissue regeneration and repair [18].

Stem cell depletion and altered intercellular communication are markers responsible for the functional deterioration associated with aging. The quantity of stem cells, as well as their ability to proliferate and differentiate can be decreased by stem cell exhaustion [12,13]. Telomere attrition, a known trigger for stem cell disturbances, can initiate mitochondrial compromise, leading to functional decline during aging, particularly in bone marrow mesenchymal stem cells (BMMSCs) [19]. In addition, studies suggest that aging reduces intestinal stem cell numbers and function, potentially linked to decreased fatty acid oxidation [20].

Intercellular communication, such as neurohormonal signaling, is impaired during aging owing to increased inflammatory response, decreased immune system resistance to pathogens and malignancies, and changes in the composition of the extracellular environment. In these conditions, damage in one tissue can cause aging-specific damage in other tissues [1,12,13]. Impaired intercellular communication also leads to inflamaging, a concept characterized by elevated levels of proinflammatory factors [3].

The progression of biological aging can be measured by assessing certain aging-related parameters [21,22], which comprise biomarkers of ‘damage’ and ‘compensation’ for various aging hallmarks. These hallmarks are reflected in molecular, physiological, pathological, and psychological changes [13,21,23], collectively contributing to clinical manifestations commonly observed in elderly individuals, including frailty, sarcopenia, anemia, nutritional deficiencies, and compromised immune function. In addition, these hallmarks are associated with age-related conditions such as cardiovascular disease, cancer, diabetes, and Alzheimer's disease [1].

Measuring metabolic parameters is a key aspect of evaluating biological aging [24]. Aging leads to a decline in metabolism and muscle mass, and an increase in fat mass, resulting in reduced energy expenditure for the body’s basal needs. The basal metabolic rate (BMR) reflects the energy required to maintain the body’s fundamental functions at rest. Adults typically experience a 1–2% decrease in BMR every 10 years. Furthermore, BMR is linked with metabolic age, which reflects the correlation between the BMR of an individual and those in the same age group. A higher BMR being associated with a lower metabolic age and vice versa [25]. Another metabolic parameter that can be analyzed is the resting metabolic rate (RMR), which represents the energy required to maintain the body’s basic functions and accounts for 60–70% of daily energy expenditure. Although RMR decreases with age, it may increase in older individuals owing to metabolic adjustments compensating for declining health and reduced functional capacity [24].

### Obesity-related early aging

Aging can progress at varying rates depending on the accumulation of damage or the decline in resistance function [13]. Obesity is an important factor contributing to accelerated aging [3]. Defined by an abnormal or excessive accumulation of body fat, obesity is most commonly assessed using the body mass index (BMI) [26]. According to the Asia Pacific classification, obesity is diagnosed when the BMI exceeds 24.9 kg/m^2^, whereas the World Health Organization (WHO) identifies obesity at a BMI greater than 30 kg/m^2^ [26,27].

Obesity and aging are linked to systemic inflammation and can be influenced by adopting a healthy lifestyle, suggesting shared cellular and molecular pathways and underlying factors. In addition, both conditions are associated with a shortened lifespan and an increased ratio of visceral to subcutaneous adipose tissue [28,29]. Similarly to aging, obesity promotes inflammatory responses, leading to subclinical inflammation and a chronic low-grade inflammatory state resulting from unresolved inflammation and prolonged stimulation [30]. Excessive macronutrient intake in obesity can lead to adipocyte hypertrophy, hypoxia, or necrosis, triggering macrophage infiltration and activating various proinflammatory pathways such as c-Jun N-terminal kinase (JNK), IkappaB kinase (IKK), protein kinase R (PKR), and toll-like receptors. These pathways may increase levels of proinflammatory cytokines such as tumor necrosis factor α (TNF-α), interleukin 6 (IL-6), and C-reactive protein (CRP), and decrease adiponectin levels [31].

Obesity can disrupt insulin signaling pathways, leading to insulin resistance, increased fat formation through triglyceride hydrolysis into fatty acids, increased sympathetic activity, and dysregulation of the renin-angiotensin-aldosterone system [31]. It is frequently associated with metabolic syndrome, characterized by low-grade inflammation marked by significantly elevated levels of inflammatory markers such as TNF-α and high mobility group box 1 (HGMB-1) [29]. This chronic systemic inflammation can lead to insulin resistance, β-cell dysfunction and ultimately type 2 diabetes, nonalcoholic fatty liver disease, retinopathy, cardiovascular disease, nephropathy, and many other age-related diseases [32–34].

**Figure 1:**
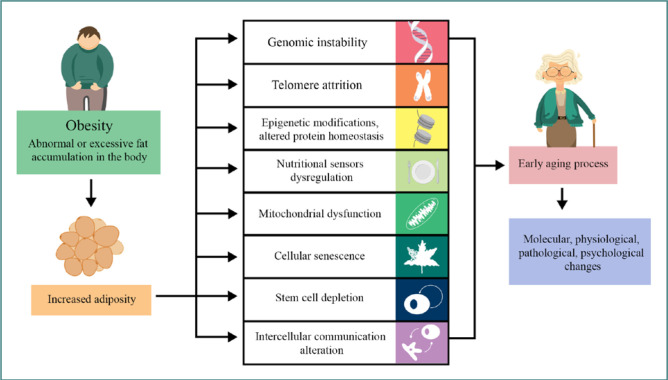
Illustration of how obesity induces the early aging process

Obesity is thought to accelerate aging and contribute to the manifestation of aging-related parameters. Figure 1 illustrates how aging can be accelerated by obesity. An accumulation of DNA damage and a decrease in DNA repair can trigger adiposity defects and affect other tissues, increase inflammation and the production of mitochondrial ROS, and reduce the level of antioxidants [35]. In addition, obesity may increase the secretion of proinflammatory mediators and decrease the production of anti-inflammatory or insulin-sensitizing factors. This imbalance can prompt adipocytes, endothelial cells, and immune cells to release proinflammatory cytokines, endothelial adhesion molecules, and pro-atherogenic and chemotactic mediators within adipose tissue. Obesity may also influence the unfolded protein response (UPR), mediated by genomic instability, accumulated DNA damage, and proteasome dysfunction [3,35].

Obesity can also cause DNA methylation and telomere shortening due to epigenetic modifications [3,36]. Telomere shortening is influenced by a variety of factors, including age, smoking, stress, gender, genetic background, nutritional status, food and alcohol intake, and physical activity. Excessive nutrition associated with obesity can induce inflammation and oxidative stress, both of which can accelerate telomere shortening [37]. A study found that obesity and subcutaneous (especially truncal) adiposity were important determinants of telomere shortening in a group of Indian women with abnormal fasting glycemia [38]. Interestingly, a major multicenter study revealed that higher levels of child obesity markers are associated with shorter telomeres [39]. The effect of obesity on epigenetics has been demonstrated by the association between BMI and accelerated epigenetic aging in a sample of children at high risk for obesity, suggesting that early-onset accelerated epigenetic aging can occur in obese individuals [40].

Obesity may accelerate aging by influencing several factors [3,41–43]. The dysregulation of nutritional signaling, mediated by the IIS, sirtuins, mTOR, and mitogen-activated protein kinase (MAPK) pathways, is a key mechanism by which obesity impacts aging [3]. Studies have shown that obesity can increase the number of senescent T cells and macrophages in the inflammatory foci of the visceral adipose tissue of obese mice [42]. Furthermore, obesity can change systemic and local microenvironments, impairing stem cell plasticity and decreasing their regenerative potential [43].

A cross-sectional cohort study involving young and middle-aged individuals found a correlation between poor metabolic health and obesity, and morphological and functional signs of aging in the brain [44]. Obesity exacerbates immunological dysfunction by accelerating immunological sensitivity, increasing inflammation, and altering immune cell function [45,46]. Together with genetic, environmental, and behavioral factors, obesity contributes to the onset of degenerative diseases, further influencing the aging process [13].

### IF, a potential anti-aging intervention

#### Definition and types of IF

IF is one of the new approaches for preventing age-associated diseases [9,47]. IF entails periods of fasting that typically last 12 h or more [11]. According to another definition, during IF, periods of fasting or minimal food intake are alternated with periods of unrestricted eating [48]. Common IF methods include alternate-day fasting (ADF), alternate-day modified fasting (ADMF), and periodic fasting (PF) [11,49,50].

The practice of IF, limited to specific time periods, is called time-restricted feeding (TRF), a term more commonly used in non-human studies, whereas in human subjects it is referred to as time-restricted eating (TRE). However, many studies use these terms interchangeably [50–55]. TRF or TRE encompasses various types, including early time-restricted eating (eTRE) and delayed time-restricted eating (dTRE) [56].

Another variant of IF is the 5:2 diet, involving modified fasting for 2 days per week [50,51]. It is important to note that different religions have distinct fasting practices, such as the Ramadan fast among Muslims, the Greek Orthodox Fast, and the Daniel Fast among Jews [9]. An overview of the different types of IF and their definitions is presented in Table 1.

**Table 1:** Types of IF and definitions

Types of IF	Definitions
TRF	Ad libitum food intake is allowed only during specified hours, creating prolonged intervals without food [50,138]
eTRE	Limits the eating window to 4–10 h (most commonly 8 h), with food consumed in the earlier part of the day, with the remaining 14–20 h in an unfed state [56]
dTRE	Limits the eating window to 4–10 h (most commonly 8 h), with food consumed in the later part of the day, with the remaining 14–20 h in an unfed state [56]
ADF	Involves a day of fasting alternated with a day with ad libitum food intake [11,49,50]
ADMF	Involves a day of fasting, with less than 25% of the normal calorie intake, alternated with a day with ad libitum food intake [11,49,50]
PF	Fasting for 2–21 days [11,49]
5:2 diet	Eating ad libitum for 5 days per week, with severely restricted calorie intake on the other 2 days, to about 25% of normal levels to maintain energy balance [50]
Religious fasting	Fasting is essential in many religious and spiritual practices, such as the Ramadan, Greek Orthodox, or the Daniel fast practiced by Jews [9,50]

#### The benefits of IF on body adaptation

Fasting entails abstaining from food for extended periods of time, ranging from several hours to several days, prompting important changes in metabolism termed as a ‘metabolic switch’. This transition typically occurs between 12 to 36 h after eating. During fasting, neurons and red blood cells still need glucose to produce adenosine triphosphate (ATP). In the first few hours of fasting, glycogen stores in the liver and muscles are broken down to provide glucose [11]. As glycogen becomes depleted, the liver–brain–adipose neurocircuitry is triggered to facilitate the use of fats, particularly triglycerides, to sustain energy under prolonged fasting [57].

Owing to the finite supply of glycogen, gluconeogenesis, which involves the breakdown of proteins and triglycerides, becomes a crucial energy source. The most significant metabolic change triggered by fasting is the increased production of ketone bodies (acetoacetic acid, β-hydroxy butyric acid (β-HB), and acetone) in the liver [58]. These ketone bodies, primarily β-HB, can freely permeate through the plasma membrane and across the blood–brain barrier, allowing them to be used as an alternative fuel for ATP synthesis and inducing mitochondrial biogenesis, particularly in neurons, heart muscle, and skeletal muscle [11,58–60].

One of the critical mechanisms of IF is its ability to induce these metabolic switches, favoring the transition from glucose to ketone bodies, particularly β-HB [11,61]. Beyond energy provision, β-HB can induce resistance to oxidative stress and inflammation, reduce insulin-dependent energy expenditure, and improve mitochondrial function and growth, DNA repair, and autophagy. These effects are mediated through the activation of cell-protective master regulators such as nuclear factor erythroid-2-related factor 2 (Nrf2), sirtuins, and AMPK, underpinning the potential of IF as an anti-aging strategy [62].

Of the various types of IF, the three most researched are ADF, TRE, and a very low-calorie diet for three consecutive days per week (4:3 IF). These three regimens can increase circulating ketone levels to varying degrees and durations. This shows that the metabolic switch is intermittently activated [11]. Another IF regimen, such as periodic fasting, can increase circulating ketone levels [49,63]. Periodic fasting for 4 to 21 days can increase acetoacetic acid significantly from baseline to the end of the fast [49].

Although the increased production of ketone bodies is central to the benefits of IF, other factors also contribute to its positive effects and requires further research [11]. Different dietary regimens that result in higher ketone bodies, such as the ketogenic diet, have different impacts on cognitive function. A study in a rat model of Alzheimer’s disease has shown that ketone production induced by the ketogenic diet and IF has different effects on the gut microbiota, disease progression, memory function, and the gut microbiome. In the study, although a ketogenic diet impaired memory function and increased the ratio of *Proteobacteria*, particularly *Enterobacteriales*, which are relatively harmful bacteria, IF showed the potential to improve memory function and foster a healthier gut microbiome, despite containing the same amount of cellulose [64].

Fasting is known to affect various physiological functions in the body. IF can also have a positive effect on multiple organs and systems, such as the brain, liver, muscles, intestines, blood, adipose tissues, and the endocrine and cardiovascular systems [11,50,65,66]. Beyond its physiological benefits, IF also influences mental health positively. For example, an 8-week ADF has been shown to reduce depression and binge eating disorder, characterized by consuming larger meals than usual within short periods and often accompanied by loss of control over eating. Despite potential negative effects, such as irritability, fasting can also lead to positive psychological experiences, such as increased feelings of appreciation, achievement, pride, and control over increased hunger and the challenge of fasting [51,67]. The benefits of IF across various organs and systems are summarized in Table 2.

**Table 2:** The effect of IF on various organ systems

Various organs or system	Function
Brain	Improved cognition, neurotropic factor production, synaptic plasticity, mitochondrial biogenesis, and resistance to injury and disease [11,50]
Cardiovascular system	Reduced blood pressure, reduced resting heart rate, increased parasympathetic tone, stress resistance, enhanced right ventricular function, upregulated glycemic control, and protected myocardium against ischemia and inflammation-induced cellular damage [11,50,66]
Lipolysis	Lipolysis, reduced leptin production, reduced inflammation [11]
Muscles	Increased insulin sensitivity, enhanced efficiency/endurance, and reduced inflammation [11]
Intestines	Enhanced motility, reduced inflammation, and enhanced intestinal stem cell function [11,118]
Liver	Glycogen depletion, ketone production, increased insulin sensitivity, and reduced lipid accumulation [11]
Blood	Elevated ketone level, reduce glucose, insulin, and leptin levels, elevated adiponectin levels, reduced inflammatory cytokines, and reduced markers of oxidative stress [11]
Endocrine	Increased growth hormone in serum, decreased IGF-I concentration, and improved glucose metabolism [50]
Immune system	Reduced the inflammatory response [50]
Kidney	Boosted renal H_2_S production [139]

#### The difficulties of applying IF

Although fasting has numerous advantages, it also has side effects similar to dietary restrictions. One of the primary side effects is hunger, caused by an adaptive response to food deprivation. During fasting, ghrelin levels increase, leading to hunger and food-seeking behavior. An imbalance between satiety and hunger hormones can lead to eating disorders such as overeating or binge eating, particularly when following a narrow eating window in IF [50]. Excessive calorie intake can affect stomach capacity, causing discomfort and affecting sleep quality [50,68]. However, a study involving 46 obese and overweight adults found that both continuous and intermittent energy restriction did not elicit compensatory appetite adaptations, including increased hunger, satiety efficiency of food or energy intake, and overall improvements in eating behavior [69].

Research suggests that certain types of IF, such as ADF and ADMF, may regulate appetite over time [51]. TRE has a similar effect to calorie restriction by influencing peripheral satiety systems involving leptin, insulin, and GLP-1. However, compared to calorie restriction, TRE does not increase ghrelin levels significantly, suggesting various implications for appetite modulation and a potential decrease in hunger following this IF approach [70]. This is supported by studies indicating that eTRE reduces mean ghrelin levels, promotes satiety, and reduces the urge to eat [71]. Another study has shown that serum levels of ghrelin, melatonin, and leptin were notably decreased during the intermittent diurnal fasting period of Ramadan, but salivary cortisol levels were unchanged compared to the pre-fasting period [72]. Nevertheless, further research is needed given the complexity of the appetite regulation system and the diverse outcomes associated with dietary changes [73].

The additional adverse effects that may occur during fasting include headache, fatigue, adrenal stress, and chills [50]. Fasting can also lead to fluctuations in sex hormone levels and fertility. Women’s reproductive hormones are particularly sensitive to changes in calorie intake, and those practicing IF may experience menstrual irregularities, metabolic issues, and early onset of menopause. Changes in women’s menstrual cycles are often attributed to the depletion of adipose depots, which releases estrogen and disrupts the menstrual cycle [50]. Conversely, fasting appears to be a beneficial approach for managing hyperandrogenism in women with polycystic ovarian syndrome (PCOS) by improving menstruation and fertility [74]. Fasting has the potential to reduce androgen hormone levels in women, although discrepancies in data warrant further investigation. Possible symptoms of fasting include dyspepsia, vertigo, nausea, muscle soreness, and diarrhea. However, a study involving 1,422 subjects has shown that fasting for 4 to 21 days is generally safe and promotes well-being. The reported complaints were mild, and most participants did not experience adverse effects [49].

#### The potential anti-aging effect of IF

IF regimens have shown pro-longevity effects in diverse species (Table 3). These effects encompass not only biomolecular and cellular, but also physiological and well-being aspects.

**Table 3:** The effect of IF on aging markers in different species

Type of IF	Sample	Aging marker	Reference
ADF	Human	Improves physiological and molecular markers of aging in healthy, non-obese individuals	[8]
Every-other-day (EOD) intermittent fasting	Human	Decreases aging-related frailty and increases renal hydrogen sulfide production in a sexually dimorphic manner	[65]
IF	Human	Fasting enhances the cognitive function in older adults	[7]
PF	Human	Associated with anticancer proteomic signature and upregulates key regulatory proteins of glucose and lipid metabolism, circadian clock, DNA repair, cytoskeleton remodeling, immune system, and cognitive function in healthy individuals	[139]
PF	Human	Safety, health improvement, and well-being	[49]
TRF and ADF	Rats	Improve the metabolic profile and redox homeostasis	[54]
eTRF	Human	Improves 24-h glucose levels and affects markers of the circadian clock, aging, and autophagy	[55]
Short-term fasting	Mice	Activates fatty acid oxidation to enhance intestinal stem cell function during homeostasis and aging	[118]
IF	Drosophila	Transcriptional regulation and physiological responses of neuronal and muscle tissues	[140]

### The effect of IF on preventing obesity-related early aging

#### IF can regulate aging phenotypes

**Figure 2:**
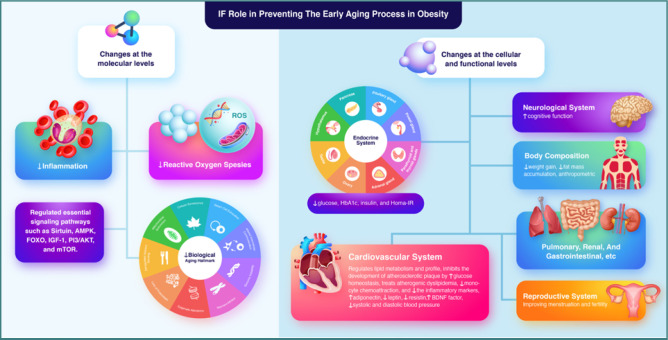
The role of IF in preventing the process of early aging in obesity through molecular and cellular mechanisms

Physiological aging involves a progressive decline in organ function [75], affecting various aspects such as body composition, the balance between energy supply and demand, neurodegeneration, and signaling networks crucial for maintaining homeostasis [22]. Specific vulnerabilities to aging can be observed across different organ systems, with the cardiovascular, endocrine, neurological, reproductive, pulmonary, renal, and musculoskeletal systems often aging more rapidly than the gastrointestinal system [75]. IF may mitigate the progression of aging in obese individuals through molecular and cellular mechanisms. A simplified representation of this concept is illustrated in Figure 2.

### The effect of IF on body composition

One of the primary aging phenotypes is characterized by changes in body composition [21,22]. Aging can induce alterations in bone, muscle, and adipose tissue. Osteosarcopenic obesity, characterized by bone and muscle loss alongside increased adiposity, is an important consequence of aging 76]. Research suggests that IF can influence body composition. For instance, aging is often associated with a decline in fat-free mass, an increase in body fat percentage, and a redistribution of fat mass, particularly towards central and visceral regions [77]. Both men and women experience an increase in body fat percentage with age, with men showing a more pronounced reduction in fat-free mass compared to women. In addition, women with stable fat-free mass tend to exhibit higher BMIs as they age [78].

Meanwhile, IF, with or without high-intensity interval training, yielded significantly less weight gain, reduced fat mass accumulation, and lowered serum levels of low-density lipoproteins (LDL) in obese mice [79]. In another study, a 30-day period of IF has improved fat metabolism, reduced fat accumulation, promoted the conversion of white fat to beige fat, and improved the composition of gut microbiota in obese mice [80]. Moreover, research suggests that TRE reduces body weight, BMI, percentage of body fat, and waist circumference, while also contributing to a decrease in cardiovascular risk [81].

Changes in body composition can have a major effect on daily energy expenditure. Total energy expenditure (TEE) may decrease with aging owing to reductions in BMR and physical activity. The estimated TEE, derived from the estimated energy requirement (EER), also called energy expenditure estimation (EEE), relies heavily on an individual’s BMR or RMR [82]. IF has been shown to influence metabolism by increasing adiponectin levels, which can further activate AMPK and PGC-1α, leading to enhanced mitochondrial biogenesis. Adiponectin also acts on the brain to increase REE, thereby contributing to weight loss [11,83,84]. Moreover, an increase in REE may also occur owing to the actions of norepinephrine [85], potentially affecting the BMI and metabolic age. However, demonstrating these effects in studies has proven challenging owing to difficulties in regulating diet, physical activity, and body composition [84,86,87].

### The effect of IF on the neurological system

Cognitive decline is an important aging phenotype within the neurological system [23]. Obesity has been linked to cognitive impairment, representing an aging marker [88–90]. Notably, a high-fat diet in young adult and middle-aged rats has been shown to promote hippocampal inflammatory responses, indicative of early aging in the brain [88]. In addition, morbid obesity has been associated with lower executive performance, including inhibitory control, verbal fluency, and psychomotor speed [91]. In a study involving individuals with mild cognitive impairment, older patients who consistently practiced IF had superior cognitive scores and improved cognitive function at the 36-month follow-up [7]. Similarly, another study reported that IF practiced from dawn to sunset over a 30-day period has improved cognitive function in healthy adults [92].

### The effect of IF on the cardiovascular system

Aging and the decline in cardiovascular system performance are closely associated [75], this process often leading to the development of cardiovascular diseases owing to changes in cardiac structure, the autonomic nervous system, and the accumulation of epigenetic changes [93]. Notably, obesity serves as a common substrate for cardiac aging and heart disease, precipitating changes in cardiovascular structure and function, inflammatory response, oxidative stress, mitochondrial dysfunction, insulin resistance, and telomere length loss through the disruption of autophagy and mitophagy [94,95]. IF has a substantial role in cardiovascular disorders by regulating lipid metabolism to reduce body mass and positively influence lipid profile parameters [96,97]. By mitigating atherogenic dyslipidemia, decreasing monocyte chemoattraction, and reducing levels of inflammatory markers such as IL-6, homocysteine, and CRP, IF can contribute to the prevention of atherosclerotic plaque formation [96,98,99]. In addition, IF has been shown to increase brain-derived neurotrophic factor (BDNF) levels, lowering systolic and diastolic blood pressure through the stimulation of the parasympathetic nervous system [96,100].

### The effect of IF on the endocrine system

Changes in the endocrine system during aging can contribute to the onset of various endocrine diseases, including osteoporosis, metabolic syndrome, type 2 diabetes mellitus, and other related conditions [101,102]. Alterations in the thyrotropic, somatotropic, adrenal, and gonadal axes, as well as disruptions in bone formation, calcium regulation, and glucose homeostasis are among the components of the human endocrine system implicated in the aging process [101]. Obese individuals often experience abnormalities in the endocrine system [103], leading to weight gain and other symptoms owing to the underlying hormonal imbalance [104]. Hypothalamic-pituitary dysfunction, encompassing issues like thyrotropic dysfunction, somatotropic dyshomeostasis, impaired gonadotropic function, and other abnormalities, is also associated with obesity [103,105,106].

Hormone replacement therapy, commonly used to treat endocrine disorders, can be controversial due to its substantial side effects [101,107]. By contrast, IF has emerged as an alternative approach for managing endocrine issues associated with aging. Research on obese individuals has shown that in addition to gradually reducing weight, BMI, glucose, HbA1c, insulin, and homa-IR levels, IF can also lower TSH and IGF-1 levels and influences GH levels [63]. The ability of IF to lower HbA1c, body weight, and total daily insulin dose suggests its potential as a beneficial treatment option for individuals with type 2 diabetes who are inadequately managed and undergoing insulin therapy [108]. In addition, IF has been shown to increase plasma adiponectin levels while decreasing leptin and resistin levels by stimulating the parasympathetic nervous system [96,100]. By regulating glucose and insulin levels, IF can contribute to the management of glycemic profiles and obesity [96,109]. According to another study, fasting may effectively address hyperandrogenism in women with PCOS by improving menstruation and fertility. However, it may reduce the level of androgens in men, potentially affecting their libido and metabolic health [74,110].

### The effect of IF on other systems

Degeneration of the pulmonary, renal, and gastrointestinal systems is also associated with aging [75,111–113]. Obesity exacerbates the risk of respiratory tract diseases owing to its mechanical and inflammatory effects. In addition, obesity increases the risk of developing various gastrointestinal conditions, including functional gastrointestinal disorders, inflammatory bowel disease, pancreatitis, and gastrointestinal cancer [113–116]. Although the effect of IF on aging and obesity in these three systems is still being explored, evidence suggests its potential benefits.

IF has shown promise in preventing and delaying the progression of diabetic nephropathy, offering potential mechanisms for reducing renal injury in diabetic nephropathy [117]. In addition, fasting has been found to activate fatty acid oxidation, enhancing intestinal stem cell function during homeostasis and aging, potentially serving as a strategy for promoting intestinal regeneration [118]. However, studies have shown that IF did not significantly change spirometric data in male patients with stable chronic obstructive pulmonary disease, nor did it affect the severity of asthma and spirometric findings in patients with moderate to severe asthma. Nevertheless, fasting can be considered safe for patients with asthma [119,120].

An intriguing finding is that age and obesity affect the risk and severity of COVID-19 [115,121]. According to a recent study, IF could serve as a supplemental therapy to reduce the risk of developing chronic diseases and may hold promise in the treatment of infectious diseases like COVID-19. Further investigation is required to determine the potential benefits of IF for respiratory system performance [122].

### Essential molecules and signaling pathways

A recent study showed that IF influences the expression of circulatory miRNAs, epigenetic modulators crucial for intercellular communication. Specifically, a TRE regimen had differential effects on circulatory miRNA expression in older overweight adults [123]. The researchers identified 14 circulatory miRNAs that were differentially expressed before and after the TRE regimen. The downregulated miRNA targets suggested increased expression of genes such as phosphatase and tensin homolog (*PTEN*), tuberous sclerosis 1 (*TSC1*), and Unc-51-like kinase 1 (*ULK1*), which may inhibit cell development and activate cell survival pathways, thereby promoting healthy aging [123].

By minimizing the generation of ROS and inflammation, IF can extend life and improve health at the cellular and molecular levels. ROS have been found to affect redox status through activating transcription factors and apoptosis. Long-term IF has been demonstrated to impact the redox state in human research, resulting in reduced lipid peroxidation, improved plasma total antioxidant capacity, and elevated uric acid levels [49]. ADF and TRE have also shown strong associations with ROS production, the activity of the erythrocyte plasma membrane redox system (PMRS), plasma protein oxidation, lipid peroxidation, IL-6, and TNF levels in mice. In addition, advanced glycation end products (AGES), glutathione, antioxidants, and protein carbonyl levels significantly decrease with ADF [54].

Cellular senescence, characterized by increased oxidative stress and inflammation, is one of the signs of aging [12]. In obesity, the increased mass of adipose tissue leads to an increase in systemic inflammation. A calorie-restricted high-fat diet (HFCR) has been found to improve glucose tolerance and lower liver triglyceride, total cholesterol, and plasma leptin/adiponectin ratio levels in rats compared to a group that only received a high-fat diet. HFCR also reduced lipid peroxidation, normalized adipocyte size and morphology, reduced fatty liver, and lowered the expression of inducible nitric oxide synthase, cyclo-oxygenase-2, Nrf2, and heme oxygenase-1 in the liver. In addition, HFCR reduced the expression of manganese superoxide dismutase (MnSOD) and C-reactive protein in adipose tissue. These findings suggest that HFCR may mitigate oxidative stress, inflammation, and adverse lipid profiles induced by a high-fat diet [124].

Aging is intricately linked to various chemicals and signaling pathways that maintain cellular homeostasis and health. Disruption of these pathways can lead to imbalanced signaling and the activation of substances that accelerate the aging of cells. Severe damage may cause apoptosis or initiate processes that contribute to tumor development [125]. Key signaling pathways involved in aging include sirtuins, AMPK, fork head box O (FOXO) transcription factors, IGF-1, phosphatidylinositol 3-kinase (PI3K)/Akt, and mTOR. Dietary interventions such as calorie restriction, protein restriction, and a low protein/high carbohydrate diet can modulate these pathways, potentially inhibiting the IIS pathway. Reduced Akt activity inhibits mTOR and activates FOXO, whereas calorie restriction can increase the cellular adenosine monophosphate (AMP)/ATP ratio and the NAD+/NADH ratio, thereby activating AMPK and sirtuins [126].

Sirtuins have a crucial role in AMPK activation through a positive feedback loop. By phosphorylating and deacetylating, AMPK and sirtuins activate FOXO and peroxisome proliferator-activated receptor gamma coactivator 1-alpha (PGC-1α), respectively. PGC-1α also activates FOXO, increasing the production of essential antioxidant enzymes and pathways involved in autophagy and mitophagy. PGC-1α also promotes the transcription of numerous critical genes involved in stress resistance, fatty acid oxidation, and mitochondrial biogenesis pathways. These interconnected pathways, facilitated by positive feedback loops including AMPK, sirtuins, FOXO, and PGC-1α, orchestrate a coordinated response, broadening the spectrum of potential health outcomes [126].

The role of IF in delaying aging in obesity involves the modulation of various signaling pathways and substances. Dietary interventions, such as IF and restriction diets, have been shown to mitigate aging by enhancing the activity of AMPK, sirtuin 1, PGC-1α, and FOXO [12]. Activating the AMPK/SIRT1 pathway in the liver and white adipose tissue can reduce obesity, improve thermogenesis, and suppress inflammation, as demonstrated in a study involving male C57BL/6 mice fed a high-fat diet to induce obesity. Following 4 weeks of short-term mild calorie restriction therapy, improvements were observed in hepatocyte steatosis, white adipogenesis, and energy expenditure, along with the increased expression of sirtuin 1, PGC-1 α, and phosphorylated AMPK in subcutaneous white adipose tissue and hepatic tissues. In addition, calorie restriction may contribute to lower body weight, total serum cholesterol, fasting blood glucose, and insulin levels by reducing nuclear factor-kappa B (NF-KB) protein levels and endothelial nitric oxide synthase (eNOS) expression [127].

The deregulation of mTOR is also a hallmark of aging. mTOR, a serine/threonine protein kinase, controls protein synthesis, cell proliferation, and cell growth. It integrates hormonal signals from the IIS pathway and signals from specific nutrients, particularly amino acids such as leucine [125]. IF is closely linked to mTOR and is recognized for its potential to extend lifespan. In healthy individuals, eTRE can modulate the expression of genes that control the circadian clock in the morning and evening while upregulating longevity genes such as mTOR and sirtuin 1 in the morning. Autophagy-related genes also exhibit increased expression in the morning and evening [55]. In a study involving obese rats fed a high-fat diet, calorie restriction was found to reverse alterations in skeletal muscle growth signaling regulators induced by obesity. Calorie restriction mitigated the effects of obesity on the lipogenic protein sterol regulatory-element binding protein 1 (SREBP1), attenuated mTORC1 hyperactivation, reduced signaling through extracellular signal-regulated protein kinases 1 and 2 (ERK1/2), and induced the expression of negative growth regulators such as regulated in development and DNA damage responses 1 (REDD1) and cleaved caspase 3 in skeletal muscle, as evidenced by Western blot analysis [128].

FOXO is a crucial regulator of metabolic homeostasis, redox balance, and stress response. It responds to various factors, including growth hormone levels, oxidative and genotoxic stress, and diminished nutritional reserves. FOXO exerts its effects by enhancing the activity of antioxidants such as MnSOD, catalase, and manganese. Moreover, FOXO-mediated activation of PGC-1α affects various cellular processes, including apoptosis, inflammation, cellular resistance to stress, proteostasis, autophagy, mitophagy, and stem cell activity [126,129–131].

Four distinct types of FOXO have been identified in mammals: FOXO1, FOXO3, FOXO4, and FOXO6. It is believed that FOXO3 is particularly implicated in the aging process. Calorie restriction has been shown to upregulate numerous anti-aging genes in human skeletal muscle by activating FOXO3, including those involved in antioxidant defense, DNA repair, and autophagy. In addition, PGC-1α, crucial in energy regulation, can interact with FOXO proteins [126]. FOXO3a promotes the expression of human telomerase reverse rtranscriptase (hTERT), an essential regulator of telomerase activity in aging, by activating c-MYC and extending the lifespan of human fibroblasts [132,133]. In differentiated 3T3-L1 adipocytes, FOXO3a may also modulate autophagy, potentially influencing lipid accumulation and inflammation in obesity [134]. Further research is required to elucidate the multifaceted roles of FOXO3a, which can both counteract aging and contribute to age-related conditions such as obesity.

### Future directions

Investigating the relationship between obesity-related premature aging and intermittent fasting (IF) remains an important area of research. Although some forms of IF have shown promise in attenuating aging and obesity, not all types of IF have been extensively studied in obese populations. ADF, for instance, has demonstrated improvements in aging-related physiological and molecular markers in healthy, non-obese adults [8]. Periodic fasting has been associated with reduced cardiovascular risk factors and substantial weight loss, including decreased obesity-related parameters such as abdominal circumference and blood pressure [49]. In addition, eTRE is able to modify autophagy indices, aging-related processes, the circadian rhythm, and 24-h glucose levels [54]. Therefore, further research is required to identify the most effective type of IF for combating early aging in obese individuals.

Individual responses to IF can vary significantly based on factors such as physiology, current health status, dietary preferences, and environmental factors. Therefore, it is essential to choose an IF regimen that takes into account each person's unique circumstances. Further sophisticated phenotypic and genotypic research is required to better understand the molecular mechanisms underlying the effects of IF on delaying aging [51]. In addition, as IF can change affect the timing and levels of hormone release, consideration should be given to any potential adverse effects when incorporating it into a dietary regimen, particularly for individuals with specific health conditions [135].

### Policy recommendation

The WHO provides comprehensive guidelines for addressing obesity, aiming to improve overall population nutrition and diet quality, as well as the governance of the food system, to ultimately enhance health and well-being. These guidelines include creating environments that promote access to nutritious food and beverages, advocating for the benefits of a healthy diet throughout life, particularly for the most vulnerable groups, and supporting health systems to promote healthy dietary patterns [136].

Furthermore, the Centers for Disease Control and Prevention (CDC) emphasizes that obesity is a multifaceted issue that requires a diversified approach. Collaboration among policy makers, state and local organizations, businesses, schools, community leaders, healthcare professionals, and individuals is essential to create an environment that encourages healthy lifestyles [137].

## CONCLUSION

Obesity can accelerate the onset of aging, manifesting through various markers such as intercellular communication, telomere attrition, dysregulated nutritional signaling, disrupted protein homeostasis, mitochondrial dysfunction, cellular senescence, and stem cell disorders. This early aging induced by obesity may predispose individuals to future degenerative diseases and health complications. Hence, there is a pressing need for simple, safe, and effective treatment options to address this issue. In recent years, IF has emerged as a promising approach with numerous benefits. IF has the potential to mitigate obesity-related early aging through its influence on molecular and cellular pathways, effectively regulating various body systems. By modulating key regulatory pathways such as sirtuins, PGC-1α, IIS, PI3K/Akt, FOXO, and mTOR, IF can help prevent the premature aging associated with obesity. Given its potential to promote healthier lifestyles and prevent obesity-related early aging, further research into the mechanisms and efficacy of IF is warranted.
